# A68 PERCEPTIONS OF ADOLESCENTS AND YOUNG ADULTS WITH INFLAMMATORY BOWEL DISEASE REGARDING A BIOPSYCHOSOCIAL TRANSITION INTERVENTION: A QUALITATIVE STUDY

**DOI:** 10.1093/jcag/gwad061.068

**Published:** 2024-02-14

**Authors:** B Allemang, M Browne, M Barwick, N Bollegala, N Fu, K Lee, A Miatello, I Nistor, E Dekker, S J Anthony, E I Benchimol

**Affiliations:** Child Health Evaluative Sciences, SickKids Research Institute, Toronto, ON, Canada; Child Health Evaluative Sciences, SickKids Research Institute, Toronto, ON, Canada; Child Health Evaluative Sciences, SickKids Research Institute, Toronto, ON, Canada; Women's College Hospital, Toronto, ON, Canada; The University of British Columbia, Vancouver, BC, Canada; Crohn's and Colitis Canada, Totonto, ON, Canada; Child Health Evaluative Sciences, SickKids Research Institute, Toronto, ON, Canada; Crohn's and Colitis Canada, Totonto, ON, Canada; Crohn's and Colitis Canada, Totonto, ON, Canada; Child Health Evaluative Sciences, SickKids Research Institute, Toronto, ON, Canada; Child Health Evaluative Sciences, SickKids Research Institute, Toronto, ON, Canada

## Abstract

**Background:**

The transition from the pediatric to adult care healthcare system for adolescents and young adults (AYAs) with inflammatory bowel disease (IBD) is complex. This group requires support to prepare for and adapt to adult care due to the manifestation of the disease, expectations for self-management, and simultaneous life transitions. To address this need, a multi-site randomized controlled trial (RCT) is being conducted to evaluate the impact of a multimodal transition intervention on functional, clinical, and psychosocial outcomes for AYAs with IBD. An embedded qualitative study explored participants’ perspectives of the intervention's acceptability and appropriateness to support the translation of findings into practice.

**Aims:**

Using in-depth individual qualitative interviews with a purposive sample of AYAs in both the intervention and control arms of the RCT, this qualitative study aimed to understand AYAs’ experiences preparing for and undergoing service transitions, developing self-management skills, and engaging with the intervention.

**Methods:**

Semi-structured interviews were held via videoconference with 21 AYAs aged 16-18 years participating in the RCT (intervention arm n=11; control arm n=10) in Toronto, Canada. Purposive sampling was used to recruit AYAs with diverse demographic and clinical characteristics at different stages of the healthcare transition. Interviews were audio-recorded, transcribed verbatim, and analyzed using an inductive approach to reflexive thematic analysis.

**Results:**

The analysis yielded four overarching themes related to 1) individual, familial, and system-level factors promoting transition readiness, 2) areas of strength and areas for growth regarding self-management skill development, 3) perceptions of the multimodal transition intervention, including barriers and facilitators to engagement, and 4) recommendations for future transition care, including refinement of the intervention. Findings to date indicate the criticality of considering AYAs’ support systems, learning styles, and communication preferences when designing and implementing transition interventions. The qualitative data analysis is ongoing, and the full results will be presented.

**Conclusions:**

The perspectives of AYAs with IBD can inform the development and refinement of clinical interventions, ensuring they are tailored to recipients’ unique needs and preferences and promote engagement and skill development during pediatric-adult transitions.

**Funding Agencies:**

CCCThe Leona M. and Harry B. Helmsley Charitable Trust

